# Erratum: Discovery of divided RdRp sequences and a hitherto unknown genomic complexity in fungal viruses

**DOI:** 10.1093/ve/veab027

**Published:** 2021-06-05

**Authors:** Yuto Chiba, Sayoko Oiki, Takashi Yaguchi, Syun-ichi Urayama, Daisuke Hagiwara

*Virus Evolution*, 2020, 7(1): veaa101

https://doi.org/10.1093/ve/veaa101

Published: 16 December 2020

In the originally published version of this manuscript, Table 1 was inadvertently omitted prior to publishing. This error has been corrected.

