# Supplementary material for: Discovery of divided RdRp sequences and a hitherto unknown genomic complexity in fungal viruses
Source: Virus Evol. 2020 Dec 16;7(1):veaa101. doi: 10.1093/ve/veaa101 (PMC7816673; doi:10.1093/ve/veaa101)
Supplement: veaa101_Supplementary_Data [file veaa101_Supplementary_Data.zip › Table_S3.docx]

**Table S3. Interactions between ORF1 and ORF2**

| Hydrogen bonds (<3.3 Å) | | | | | | |
| --- | --- | --- | --- | --- | --- | --- |
| ORF1 | amino acid residue | atom | ORF2 | Amino acid residue | atom | distance (Å) |
|  | 484(SER) | OG [O] |  | 58(GLY) | O [O] | 2.84 |
|  | 500(SER) | OG [O] | motif D | 103(ASN) | N [N] | 3.17 |
|  | 500(SER) | OG [O] | motif D | 104(PHE) | N [N] | 3.14 |
|  | 512(GLU) | O [O] | motif D | 100(ARG) | NH1 [N] | 2.73 |
|  | 515(GLU) | OE1 [O] |  | 50(LYS) | NZ [N] | 2.62 |
|  | 516(SER) | O [O] |  | 50(LYS) | NZ [N] | 2.71 |
|  | 517(SER) | O [O] |  | 69(SER) | N [N] | 3.18 |
|  | 519(LEU) | N [N] |  | 67(GLY) | O [O] | 2.74 |
| motif A | 520(SER) | O [O] | motif D | 96(SER) | N [N] | 3.04 |
| motif A | 520(SER) | OG [O] | motif D | 103(ASN) | N [N] | 3.1 |
| motif A | 520(SER) | OG [O] | motif D | 103(ASN) | O [O] | 3.11 |
| motif A | 521(THR) | O [O] | motif C | 65(GLU) | N [N] | 3.19 |
| motif A | 521(THR) | N [N] | motif C | 65(GLU) | O [O] | 2.77 |
| motif A | 523(TRP) | NE1 [N] | motif C | 65(GLU) | OE2 [O] | 2.72 |
|  | 600(HIS) | ND1 [N] | motif C | 62(GLY) | O [O] | 2.94 |
|  | 601(LEU) | N [N] |  | 60(GLU) | OE2 [O] | 3.06 |
|  | 612(GLN) | OE1 [O] |  | 5(ARG) | NE [N] | 2.88 |
|  | 613(ASP) | OD2 [O] |  | 52(GLU) | N [N] | 2.94 |
|  |  |  |  |  |  |  |
| van der Waals (C-C distance <4.5 Å) | | | | | | |
| ORF1 | Amino acid residue | atom | ORF2 | Amino acid residue | atom | distance (Å) |
|  | 518(VAL) | C [C] |  | 694(VAL) | C [C] | 4.37 |
