# Supplementary material for: Discovery of divided RdRp sequences and a hitherto unknown genomic complexity in fungal viruses
Source: Virus Evol. 2020 Dec 16;7(1):veaa101. doi: 10.1093/ve/veaa101 (PMC7816673; doi:10.1093/ve/veaa101)
Supplement: veaa101_Supplementary_Data [file veaa101_Supplementary_Data.zip › Table S1 (155 strains).pdf]

Table S1. Fungal species and strains used in this study

Species	Strain	Origin	Sequenced by FLDS as isolate or pooled library	Presence of viruses
Aspergillus fumigatus	IFM 62632	Coffee beans	Isolate	AfuPmV1, AfuNSRV1
Aspergillus fumigatus	IFM 63147	Paranasal sinus	Isolate	AfuCV, AfuNV2, AfuBOV1
Aspergillus fumigatus	IFM 63431	Lung abscess	Isolate	AfuNV2
Aspergillus fumigatus	IFM 64916	Sputum	Isolate	AfuBOV1
Aspergillus fumigatus	IFM 62347	Soil	pool 1	No
Aspergillus fumigatus	IFM 62354	Soil	pool 1	No
Aspergillus fumigatus	IFM 62500	Soil	pool 1	No
Aspergillus fumigatus	IFM 62355	Soil	pool 1	AfuMV1
Aspergillus fumigatus	IFM 62503	Soil	pool 1	No
Aspergillus fumigatus	IFM 62504	Soil	pool 1	No
Aspergillus fumigatus	IFM 62497	Soil	pool 1	No
Aspergillus fumigatus	IFM 62498	Soil	pool 1	No
Aspergillus fumigatus	IFM 62507	Soil	pool 1	No
Aspergillus fumigatus	IFM 62346	Soil	pool 1	No
Aspergillus fumigatus	IFM 62505	Soil	pool 1	No
Aspergillus fumigatus	IFM 62502	Soil	pool 1	No
Aspergillus fumigatus	IFM 62345	Soil	pool 1	No
Aspergillus fumigatus	IFM 62501	Soil	pool 1	No
Aspergillus fumigatus	IFM 62506	Soil	pool 1	No
Aspergillus fumigatus	IFM 62629	Soil	pool 1	AfuNV2
Aspergillus fumigatus	IFM 62628	Soil	pool 1	No
Aspergillus fumigatus	IFM 62630	Soil	pool 1	No
Aspergillus fumigatus	IFM 62508	Soil	pool 1	No
Aspergillus fumigatus	IFM 62997	Sputum	pool 1	No
Aspergillus fumigatus	IFM 63141	Fungal ball	pool 2	No
Aspergillus fumigatus	IFM 62944	Abscess	pool 2	No
Aspergillus fumigatus	IFM 63146	Maxillary sinus	pool 2	No
Aspergillus fumigatus	IFM 63181	Respiratory secretion	pool 2	No
Aspergillus fumigatus	IFM 62942	Sputum	pool 2	No
Aspergillus fumigatus	IFM 63172	Sputum	pool 2	No
Aspergillus fumigatus	IFM 63399	BALF	pool 2	No
Aspergillus fumigatus	IFM 63397	Sputum	pool 2	No
Aspergillus fumigatus	IFM 63265	Sputum	pool 2	No
Aspergillus fumigatus	IFM 63173	Sputum	pool 2	No
Aspergillus fumigatus	IFM 63338	Sputum	pool 2	No
Aspergillus fumigatus	IFM 63398	Sputum	pool 2	No
Aspergillus fumigatus	IFM 63296	Eye abscess	pool 2	No
Aspergillus fumigatus	IFM 63439	Sputum	pool 2	AfuRV1
Aspergillus fumigatus	IFM 63437	Paranasal sinus	pool 2	No
Aspergillus fumigatus	IFM 64310	Sputum	pool 2	No
Aspergillus fumigatus	IFM 64309	BALF	pool 2	No
Aspergillus fumigatus	IFM 64194	BALF	pool 2	No
Aspergillus fumigatus	IFM 63441	Sputum	pool 2	No
Aspergillus fumigatus	IFM 63440	Sputum	pool 2	No
Aspergillus fumigatus	IFM 63438	Sputum	pool 3	No
Aspergillus fumigatus	IFM 63400	Sputum	pool 3	No
Aspergillus fumigatus	IFM 64313	Sputum	pool 3	No
Aspergillus fumigatus	IFM 64451	BALF	pool 3	No
Aspergillus fumigatus	IFM 64463	Sputum	pool 3	No
Aspergillus fumigatus	IFM 64470	Sputum	pool 3	No
Aspergillus fumigatus	IFM 64452	Sputum	pool 3	No
Aspergillus fumigatus	IFM 64433	Sputum	pool 3	No
Aspergillus fumigatus	IFM 64464	Sputum	pool 3	No
Aspergillus fumigatus	IFM 64363	Lung	pool 3	No
Aspergillus fumigatus	IFM 64312	Sputum	pool 3	No
Aspergillus fumigatus	IFM 64311	Sputum	pool 3	No
Aspergillus fumigatus	IFM 64752	BALF	pool 3	No
Aspergillus fumigatus	IFM 64737	BALF	pool 3	No
Aspergillus fumigatus	IFM 64736	BALF	pool 3	No
Aspergillus fumigatus	IFM 64958	Sputum	pool 3	No

Aspergillus fumigatus	IFM 64509	Lung	pool 3	No
Aspergillus fumigatus	IFM 64507	Sputum	pool 3	No
Aspergillus fumigatus	IFM 64513	Sputum	pool 4	No
Aspergillus fumigatus	IFM 64506	Sputum	pool 4	No
Aspergillus fumigatus	IFM 64643	Sputum	pool 4	No
Aspergillus fumigatus	IFM 64503	Sputum	pool 4	No
Aspergillus fumigatus	IFM 64516	Sputum	pool 4	No
Aspergillus fumigatus	IFM 64510	Sputum	pool 4	No
Aspergillus fumigatus	IFM 64488	Sputum	pool 4	No
Aspergillus fumigatus	IFM 64508	Sputum	pool 4	No
Aspergillus fumigatus	IFM 64645	Sputum	pool 4	No
Aspergillus fumigatus	IFM 64980	Toe	pool 4	No
Aspergillus fumigatus	IFM 64863	Sputum	pool 4	No
Aspergillus fumigatus	IFM 64779	Sputum	pool 4	AfuBOV1
Aspergillus fumigatus	IFM 64780	Sputum	pool 4	No
Aspergillus fumigatus	IFM 64660	Sputum	pool 4	No
Aspergillus fumigatus	IFM 64791	Sputum	pool 4	No
Aspergillus fumigatus	IFM 64757	BALF	pool 4	No
Aspergillus fumigatus	IFM 63245	Sputum	pool 7	No
Aspergillus pseudoviridinutans	IFM 64962	BALF	pool 4	No
Aspergillus pseudoviridinutans	IFM 62839	Soil	pool 4	No
Aspergillus pseudoviridinutans	IFM 62840	Soil	pool 4	No
Aspergillus pseudoviridinutans	IFM 64769	Soil	pool 4	No
Aspergillus pseudoviridinutans	IFM 63250	Paranasal sinus (cat)	pool 5	No
Aspergillus pseudoviridinutans	IFM 59502	Eye discharge	pool 5	ApvBOV1
Aspergillus pseudoviridinutans	IFM 59503	Cornea	pool 5	ApvBOV1
Aspergillus pseudoviridinutans	IFM 61378	Cornea	pool 5	ApvBOV1
Aspergillus pseudoviridinutans	IFM 61377	Cornea	pool 5	ApvBOV1
Aspergillus pseudoviridinutans	IFM 61579	Lung	pool 5	No
Aspergillus pseudoviridinutans	IFM 60053	Femoral neoplasm	pool 5	No
Aspergillus pseudoviridinutans	IFM 62841	Soil	pool 5	No
Aspergillus pseudoviridinutans	IFM 55266	Lung	pool 5	No
Aspergillus pseudoviridinutans	IFM 54303	Sputum	pool 5	No
Aspergillus pseudoviridinutans	IFM 60685	Trachea	pool 7	No
Aspergillus udagawae	IFM 62206	Sputum	pool 5	No
Aspergillus udagawae	IFM 62180	BALF	pool 5	No
Aspergillus udagawae	IFM 58400	Sputum	pool 5	No
Aspergillus udagawae	IFM 58028	Lung	pool 5	No
Aspergillus udagawae	IFM 62171	Cornea	pool 5	No
Aspergillus udagawae	IFM 57852	Sputum	pool 5	No
Aspergillus udagawae	IFM 51744	BALF	pool 5	No
Aspergillus udagawae	IFM 5058	Eye	pool 5	No
Aspergillus udagawae	IFM 59990	Lung drainage	pool 5	No
Aspergillus udagawae	IFM 62208	Sputum	pool 5	No
Aspergillus udagawae	IFM 64644	Drainage	pool 6	No
Aspergillus udagawae	IFM 64770	Soil	pool 6	No
Aspergillus udagawae	IFM 64766	Soil	pool 6	No
Aspergillus udagawae	IFM 64768	Soil	pool 6	No
Aspergillus udagawae	IFM 64505	Sputum	pool 6	No
Aspergillus udagawae	IFM 64504	Sputum	pool 6	No
Aspergillus udagawae	IFM 64767	Soil	pool 6	No
Aspergillus udagawae	IFM 64771	Soil	pool 6	No
Aspergillus udagawae	IFM 63307	Lung	pool 7	No
Aspergillus lentulus	IFM 64004	Sputum	Isolate	AleNdsRV1
Aspergillus lentulus	IFM 64003	Sputum	Isolate	AleNdsRV1
Aspergillus lentulus	IFM 63547	Sputum	Isolate	AleNV1
Aspergillus lentulus	IFM 62627	Soil	Isolate	AlePV1
Aspergillus lentulus	IFM 65052	Lung	Isolate	AleTV1
Aspergillus lentulus	IFM 62877	Fungal ball	pool 3	No
Aspergillus lentulus	IFM 62875	Fungal ball	pool 3	No
Aspergillus lentulus	IFM 62135	Sputum	pool 6	No
Aspergillus lentulus	IFM 62177	Diaphragmatic tumor	pool 6	No

Aspergillus lentulus	IFM 61591	Sputum	pool 6	No
Aspergillus lentulus	IFM 62631	Soil	pool 6	No
Aspergillus lentulus	IFM 62136	BALF	pool 6	No
Aspergillus lentulus	IFM 62658	Eye	pool 6	No
Aspergillus lentulus	IFM 62878	Fungal ball	pool 6	No
Aspergillus lentulus	IFM 62874	Fungal ball	pool 6	No
Aspergillus lentulus	IFM 64777	Sputum	pool 6	No
Aspergillus lentulus	IFM 63459	Sputum	pool 6	No
Aspergillus lentulus	IFM 62699	Sputum	pool 6	No
Aspergillus lentulus	IFM 62876	Fungal ball	pool 6	No
Aspergillus lentulus	IFM 63728	Sputum	pool 7	No
Aspergillus lentulus	IFM 47063	Sputum	pool 7	No
Aspergillus lentulus	IFM 47457	BALF	pool 8	No
Aspergillus lentulus	IFM 54822	Lung endocrine	pool 8	No
Aspergillus lentulus	IFM 58399	Sputum	pool 8	No
Aspergillus lentulus	IFM 60648	Sputum	pool 8	No
Aspergillus lentulus	IFM 61392	Lung	pool 8	No
Neosartorya fischeri	IFM 47022	Soil	pool 7	No
Neosartorya fischeri	IFM 47146	House Dust	pool 7	No
Neosartorya fischeri	IFM 47148	House Dust	pool 7	No
Neosartorya fischeri	IFM 47147	House Dust	pool 7	No
Neosartorya fischeri	IFM 47144	House Dust	pool 7	No
Neosartorya fischeri	IFM 47141	House Dust	pool 7	No
Neosartorya fischeri	IFM 50773	Japanese pickles	pool 7	No
Neosartorya fischeri	IFM 54311	Soil	pool 7	No
Neosartorya fischeri	IFM 61637	Soil	pool 7	No
Neosartorya fischeri	IFM 47143	House Dust	pool 7	No
Neosartorya fischeri	IFM 52629	Starch	pool 7	No
Neosartorya fischeri	IFM 47142	House Dust	pool 7	No
Neosartorya fischeri	IFM 58396	Soil	pool 7	No
Neosartorya fischeri	IFM 47145	House Dust	pool 7	No
Neosartorya fischeri	IFM 52630	Starch	pool 7	No
