# Supplementary material for: Discovery of divided RdRp sequences and a hitherto unknown genomic complexity in fungal viruses
Source: Virus Evol. 2020 Dec 16;7(1):veaa101. doi: 10.1093/ve/veaa101 (PMC7816673; doi:10.1093/ve/veaa101)
Supplement: veaa101_Supplementary_Data [file veaa101_Supplementary_Data.zip › Table S2 (RNA viruses)a.pdf]

Table S2: List of the RNA viruses obtained from *Aspergillus* species in this study

Virus name	Genome (size in nt) [*2]	Number of ORF	Function of the ORF [*4]	Viral sequence with the highest hit to the found ORF	Identity [*5]	Family name of the hit virus
**Aspergillus fumigatus polymycovirus 1 (AfuPmV1)**	RNA1 (2402)	1	RdRp	Aspergillus fumigatus polymycovirus 1	**96.59%**	*Polymycoviridae* [*6]
	RNA2 (2232)	1	Protease	Aspergillus fumigatus polymycovirus 1	**98.84%**	
	RNA3 (1970)	1	MT	Aspergillus fumigatus polymycovirus 1	**98.39%**	
	RNA4 (1130)	1	PAS-rp	Aspergillus fumigatus polymycovirus 1	**97.49%**	
**Aspergillus fumigatus negative-stranded RNA virus 1 (AfuNSRV1)** [*1]	RNA1 (6455)[*3]	1	RdRp	Penicillium discovirus	69.91%	*Betamycobunyaviridae* [*6]
**Aspergillus fumigatus chrysovirus (AfuCV)**	RNA1 (3562)	1	RdRp	Aspergillus fumigatus chrysovirus	**97.94%**	*Chrysoviridae*
	RNA2 (3167)	1	CP	Aspergillus fumigatus chrysovirus	**95.59%**	
	RNA3 (3126)	1	HP	Aspergillus fumigatus chrysovirus	**96.05%**	
	RNA4 (2866)	1	HP	Aspergillus fumigatus chrysovirus	**98.11%**	
**Aspergillus fumigatus narnavirus 2 (AfuNV2)**	RNA1 (2008)	1	RdRp	Aspergillus fumigatus narnavirus 2	**97.17%**	*Narnaviridae*
	RNA2 (2118)	1	Unknown	Plasmopara viticola associated narnavirus 33	23.94%	*Narnaviridae*
	RNA3 (1301)	2 or 3	Unknown	Not found	-	-
**Aspergillus fumigatus botourmiavirus 1 (AfuBOV1)** [*1]	RNA1 (2400)	1	RdRp	Cladosporium cladosporioides ourmia-like virus 1	40.22%	*Botourmiaviridae*
**Aspergillus fumigatus mitovirus 1 (AfuMV1)**	RNA1 (2519)	1	RdRp	Aspergillus fumigatus mitovirus 1	**98.96%**	*Narnaviridae*
**Aspergillus fumigatus RNA virus 1 (AfuRV1)** [*1]	RNA1 (3611)	1	Putative RdRp	Luckshill virus	32.70%	Unclassified
	RNA2 (3447)	1	HP	Cyril virus	32.20%	Unclassified
	RNA3 (1943)	1	Unknown	Not found	-	-
**Aspergillus lentulus partitivirus 1 (AlePV1)** [*1]	RNA1 (1820)	1	RdRp	Aspergillus fumigatus partitivirus 2	84.12%	*Partitiviridae*
	RNA2 (1646)	1	CP	Aspergillus fumigatus partitivirus 2	69.75%	*Partitiviridae*
**Aspergillus lentulus narnavirus 1 (AleNV1)** [*1]	RNA1 (3071)	1	RdRp	Beihai narna-like virus 21	32.51%	*Narnaviridae*
	RNA2 (1814)	2	Unknown	Not found	-	-
**Aspergillus lentulus non-segmented dsRNA virus 1 (AleNdsRV1)** [*1]	RNA1 (2907)	2	RdRp	Beauveria bassiana RNA virus 1	65.06%	Unclassified
**Aspergillus lentulus totivirus 1 (AleTV1)** [*1]	RNA1(5171)	2	RdRp	Aspergillus homomorphus totivirus 1	76.51%	*Totiviridae*
			CP	Aspergillus homomorphus totivirus 1	83.70%	*Totiviridae*
**Aspergillus pseudoviridinutans botourmiavirus 1 (ApvBOV1)** [*1]	RNA1 (2899)	1	RdRp	Cladosporium cladosporioides ourmia-like virus 1	38.57%	*Botourmiaviridae*

*1 : A tentative name is provided.

*2 : In case the viral sequences were detected in multiple fungal strains, the representative size of viral sequence is shown.

*3 : The terminal sequence was not determined with high reliability. The presumed size is shown.

*4 : CP: coat protein; MT: methyl transferase; HP: hypothetical protein; PAS-rp: PAS (proline, alanine, and serine)-rich protein

*5 : Sequence identity scores > 90% are shown in bold.

*6 : The viral family name is suggested.
