# Supplementary material for: Discovery of divided RdRp sequences and a hitherto unknown genomic complexity in fungal viruses
Source: Virus Evol. 2020 Dec 16;7(1):veaa101. doi: 10.1093/ve/veaa101 (PMC7816673; doi:10.1093/ve/veaa101)
Supplement: veaa101_Supplementary_Data [file veaa101_Supplementary_Data.zip › Table S4 (Accession).pdf]

Table S4. List of accession numbers and the full virus names used for sequence alignments and the phylogenetic analysis

Viral family	Abbreviation	Virus name	Accession no.
Tymoviridae	TYMV	Turnip yellow mosaic virus	NP_663297.1
	OkMV	Okra mosaic virus	YP_001285472.1
	AnplV	Andean potato latent virus	YP_007517180.1
	ObwV	Oat blue dwarf virus	NP_044447.1
	MrfV	Maize rayado fino virus	NP_115454.1
	GfV	Grapevine fleck virus	NP_542612.1
	BMLV	Bee Macula-like virus	YP_009160324.1
Closteroviridae	CNFV-YN	Carnation necrotic fleck virus	YP_009506332.1
			YP_009506331.1
	CYLV-DE	Carrot yellow leaf virus	YP_003075965.1
			YP_003075964.1
	CTV-T30	Citrus tristeza virus	NP_733947.1
	GLaV-2-PN	Grapevine leafroll-associated virus 2	YP_337912.1
			YP_337911.1
	MV-1-454_004	Mint virus 1	YP_224091.1
			YP_224291.1
	GLRaV-1	Grapevine leafroll-associated virus 1	YP_004940642.1
			YP_004940641.1
	GLRaV-3	Grapevine leafroll-associated virus 3	NP_813795.3
	GLRaV-5	Grapevine leafroll-associated virus 5	YP_004901686.1
	LChV-2	Little cherry virus 2	AAP87783.1
	PMWaV-1	Pineapple mealybug wilt-associated virus 1	AAL66708.2
	PMWaV-2	Pineapple mealybug wilt-associated virus 2	AAG13938.1
	PMWaV-3	Pineapple mealybug wilt-associated virus 3	ABD62347.2
	PBNSPaV	Plum bark necrosis stem pitting-associated virus	YP_001552324.1
	BYDV-Bn03	Bean yellow disorder virus	YP_001816770.1
	BPYV-MD	Beet pseudoyellows virus	NP_940785.1
	CYV	Cucumber yellows virus	BAC66371.1
	BYVaV-SC	Blackberry yellow vein-associated virus	AAV40963.1
	CYSDV-ES	Cucurbit yellow stunting disorder virus	CAD61026.2
	LCV-CA	Lettuce chlorosis virus	ACQ82504.1
	LIYV-CA	Lettuce infectious yellows virus	AAA61797.1
	PYVV-Peru	Potato yellow vein virus	YP_054415
	SpaV-M1	Strawberry pallidosis-associated virus	YP_025082.2
	SPCSV-EA	Sweet potato chlorotic stunt virus	NP_689396.1
	ToCV-FL	Tomato chlorosis virus	YP_293695.1
	TICV-CA	Tomato infectious chlorosis virus	YP_003204952.1
Virgaviridae	TMV	Tobacco mosaic virus	NP_597746.1
	ObPV	Obuda pepper virus	BAA02700.1
	ORSV	Odontoglossum ringspot virus	CAA57642.1
	WMoV	Wasabi mottle virus	BAB82440.1
	HLSV	Hibiscus latent Singapore virus	AAQ03050.2
	CGMMV	Cucumber green mottle mosaic virus	BAA18895.1
	BPMV	Bell pepper mottle virus	ABC87810.1
	RiMV	Ribgrass mosaic virus	AEI69660.1
	CMMV	Cactus mild mottle virus	YP_002455905.1
	PeCV	Peanut clump virus	NP_620047.1
	SCSV	Sorghum chlorotic spot virus	NP_659020.2
	TRV	Tobacco rattle virus	AAD48027.2
	BSMV	Barley stripe mosaic virus	AAA46336.1

	IPCV	Indian peanut clump virus	NP_835282.1
	CWMV	Chinese wheat mosaic virus	CAB41770.1
	PMTV	Potato mop-top virus	CAB58364.1
	BSBV	Beet soil-borne virus	CAB10764.1
			NP_612629.1
	GORV	Gentian ovary ringspot virus	BAP18642.1
	PeBV	Pea early-browning virus	NP_049325.1
Bromoviridae	AMV-425	Alfalfa mosaic virus	YP_053235
			NP_041192.1
	PZSV-tomato	Pelargonium zonate spot virus	NP_619771
			NP_619770.1
	BMV-RU	Brome mosaic virus	NP_041197
			NP_041196.1
	CMV	Cucumber mosaic virus	BAA00263.1
			BAW81749.1
	OLV2	Olive latent virus 2	CAA64073.1
			NP_620042.1
	TSV	Tobacco streak virus	AAB48983
			NP_620772.1
	AGLV	Ageratum latent virus	AGN29718.1
			AGN29717
	ALMMV	Amazon lily mild mottle virus	YP_006576519.1
			BAM34543.1
	BBMV	Broad bean mottle virus	NP_658999.1
			AAA42740.1
	BSV	Blueberry shock virus	YP_008519304.1
			YP_008519305
Benyviridae	BNYVV-A-Jap	Beet necrotic yellow vein virus	NP_612615.1
	BSBMV	Beet soil-borne mosaic virus	AEK48983.1
Unclassified (viga-like viruses)	MpTLV	Macrophomina phaseolina tobamo-like virus	YP_009109559.1
	PpTlV1	Podosphaera prunicola tobamo-like virus	ATS94406.1
	ArTLV1	Acidomyces richmondensis tobamo-like virus 1	AZT88674.1
	HVLV17	Hubei virga-like virus 17	YP_009337715.1
	LuV	Luckshill virus	AWA82251.1
	Haemonchus contortus	Haemonchus contortus	CDJ82925.1
	BCCV1	Beihai charybdis crab virus 1	YP_009333242.1
	Eutypa lata UCREL1	Eutypa lata UCREL1	EMR61648.1
	Cyril virus	Cyril virus	AWA82277.1
	NB	Nippostrongylus brasiliensis	VDL70932.1
Unclassified	RsPSRV-Illinois1	Rhizoctonia solani positive-strand RNA virus 1	AMM45289.1
Narnaviridae (*Narnavirus*)	AtNV1	Alternaria tenuissima narnavirus 1	QDB74997.1
	NpNV2	Neofusicoccum parvum narnavirus 2	QDB74995.1
	AfuNV2	Aspergillus fumigatus narnavirus 2	AXE72934.1
	AfuNV1	Aspergillus fumigatus narnavirus 1	AXE72933.1
	FuPNV2	Fusarium poae narnavirus 2	YP_009272903.1
	CtNV1	Cladosporium tenuissimum narnavirus 1	QDB74996.1
	NpNV1	Neofusicoccum parvum narnavirus	QDB74994.1
	BlNV1	Blechmonas luni narnavirus 1	YP_009553634.1
	BlWNV1	Blechomonas wendygibsoni narnavirus 1	YP_009552755.1
	ScNV-20S	Saccharomyces 20S RNA narnavirus	AAC98925.1
	ScNV-23S	Saccharomyces 23S RNA narnavirus	AAC98708.1
	NarEnv	Narnaviridae environmental sample	AJT39597.1

	Lasius niger	Lasius niger	KMQ87490.1
	ONLV1	Ochlerotatus-associated narna-like virus 1	AGW51766.2
	PiRV4	Phytophthora infestans RNA virus 4	AMR08648.1
	BNLV21	Beihai narna-like virus 21	YP_009333140.1
	Sherlock virus	Sherlock virus	QED21500.1
	BBV10	Beihai barnacle virus 10	YP_009333179.1
	WNLV1	Wilkie narna-like virus 1	YP_009388589.1
	LepseyNLV1	Leptomonas seymouri RNA virus	ASN64762.1
	PserNV1	Phytomoas serpens narnavirus 1	AUF41955.1
	FuPNV1	Fusarium poae narnavirus 1	YP_009272902.1
	PvaNV32	Plasmopara viticola associated narnavirus 32	QIR30311.1
	PvaNV33	Plasmopara viticola associated narnavirus 33	QIR30312.1
	PvaNV35	Plasmopara viticola associated narnavirus 35	QIR30314.1
	MaRNAV1	Matryoshka RNA virus 1	QGV56801.1
Botourmiaviridae	EcV	Epirus cherry virus	YP_002019754.1
	OumV	Ourmia melon virus	YP_002019757.1
