# Supplementary material for: Discovery of divided RdRp sequences and a hitherto unknown genomic complexity in fungal viruses
Source: Virus Evol. 2020 Dec 16;7(1):veaa101. doi: 10.1093/ve/veaa101 (PMC7816673; doi:10.1093/ve/veaa101)
Supplement: veaa101_Supplementary_Data [file veaa101_Supplementary_Data.zip › Table S5 (primer)3a.pdf]

Table S5. List of primers for RT-PCR.

Target virus	Primer name	sequence (5'-3')	expected size of DNA fragment in bp
Aspergillus fumigatus mitovirus 1	Pool1_AfuMV1_RNA1_p8_f	AAGTCGACCGATTCGCGGCA	535
	Pool1_AfuMV1_RNA1_p8_r	GGCCACATCCAGGCCCACTC	
Aspergillus fumigatus narnavirus 2	Pool1_AfuNV2_RNA1_p1_f	AACTGGAGGCATCCAGGCGT	562
	Pool1_AfuNV2_RNA1_p1_r	TGACGTGGAGCAGTCAGTGC	
	AfuNV2_RNA1_p3_f	TGGGAACAACCGAGTCAAGG	619
	AfuNV2_RNA1_p3_r	ACCCCTAGCCGGTATAGCAA	
	AfuNV2_RNA2_p3_f	GAGAGTAATGTGGGAGGTGT	626
	AfuNV2_RNA2_p3_r	GGAGCGAAGGTTCTACCCCG	
	AfuNV2_RNA1&2_f	GGTACCGTGAAACAGTGCTG	560
	AfuNV2_RNA1&2_r	GAAGGCTTCCACCTTCTTCG	
Aspergillus fumigatus RNA virus 1	Pool2_AfuRV1_RNA1_p3_f	TTGCCGCTTCGGGCTGCTCG	645
	Pool2_AfuRV1_RNA1_p3_r	CGCGTAGTACCACATCCTGC	
	AfuRV1_RNA1_3end_p3	ACTGATTATGTGTCGGCGGG	503
	AfuRV1_RNA2_3end_p2	TCAGCCCGCAACATGTCGTG	591
Aspergillus fumigatus Botourmia virus 1	Pool4_AfuBOV1_RNA1_p1_f	CCCATCAACGCCACCTGACG	486
	Pool4_AfuBOV1_RNA1_p1_r	ACCTCACCACTCCGGGCTTC	
Aspergillus pseudoviridinutans Botourmia virus 1	Pool5_ApvBOV1_RNA1_p3_f	GTATGGTCTCCACCTGCCGC	645
	Pool5_ApvBOV1_RNA1_p3_r	ACTTCGCCCTCCACAGGAGT	
	Pool5_ApvBOV1_RNA1_p7_f	CCGGTTTCCGTGGCTGCTAA	450
	Pool5_ApvBOV1_RNA1_p7_r	CCTACGCTCAGACGCAACCC	
Aspergillus fumigatus negative-stranded RNA virus 1	AfuNSRV1_P2_F	AGGGCATTGGGGGAAAACAA	418
	AfuNSRV1_P2_R	CCTCGGATTCGCTGACTTGA	
