# Supplementary figures and images for: Discovery of divided RdRp sequences and a hitherto unknown genomic complexity in fungal viruses
Source: Virus Evol. 2020 Dec 16;7(1):veaa101. doi: 10.1093/ve/veaa101 (PMC7816673; doi:10.1093/ve/veaa101)

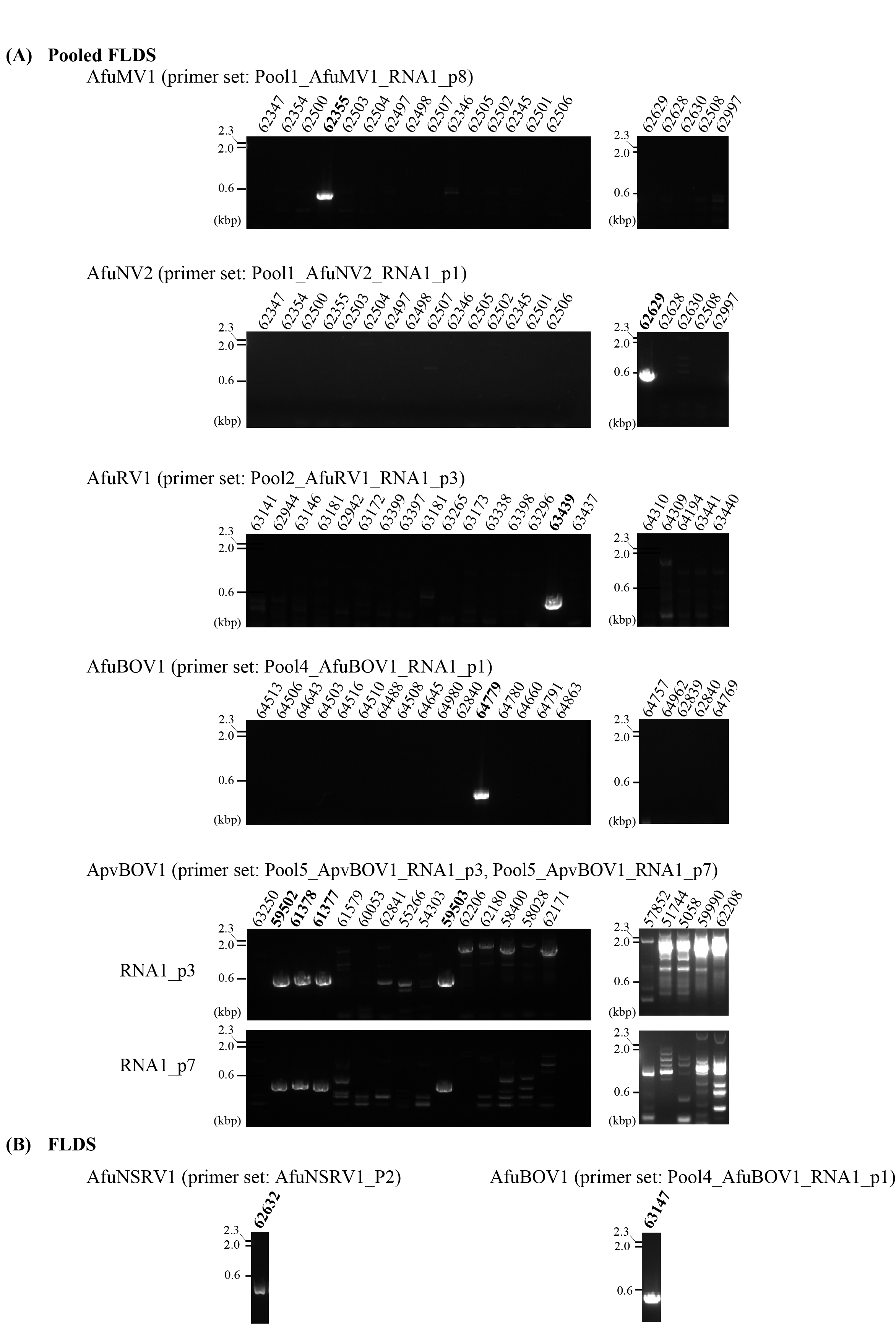

Supplement: veaa101_Supplementary_Data [file veaa101_Supplementary_Data.zip › Fig. S1.jpg]

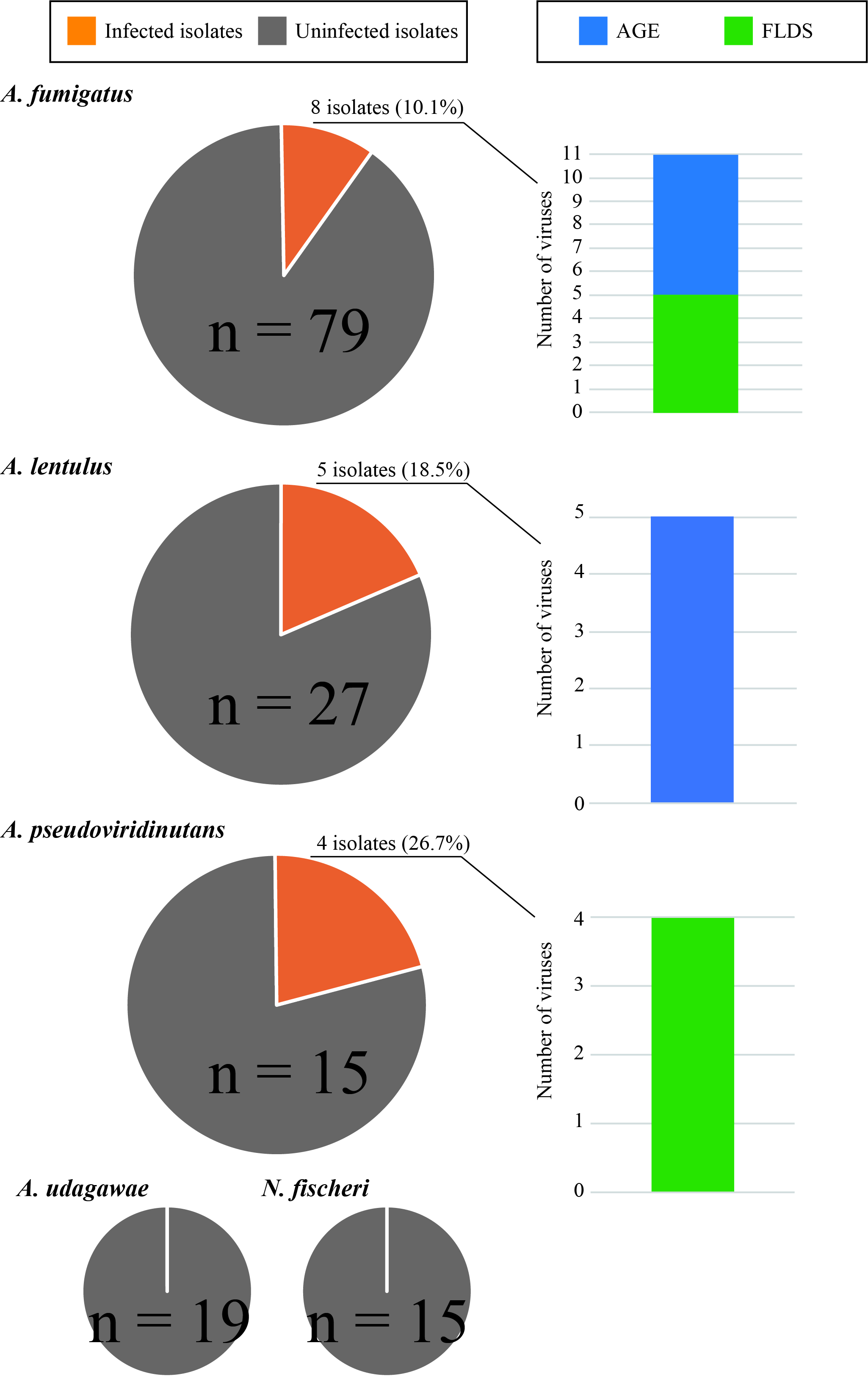

Supplement: veaa101_Supplementary_Data [file veaa101_Supplementary_Data.zip › Fig. S2.jpg]

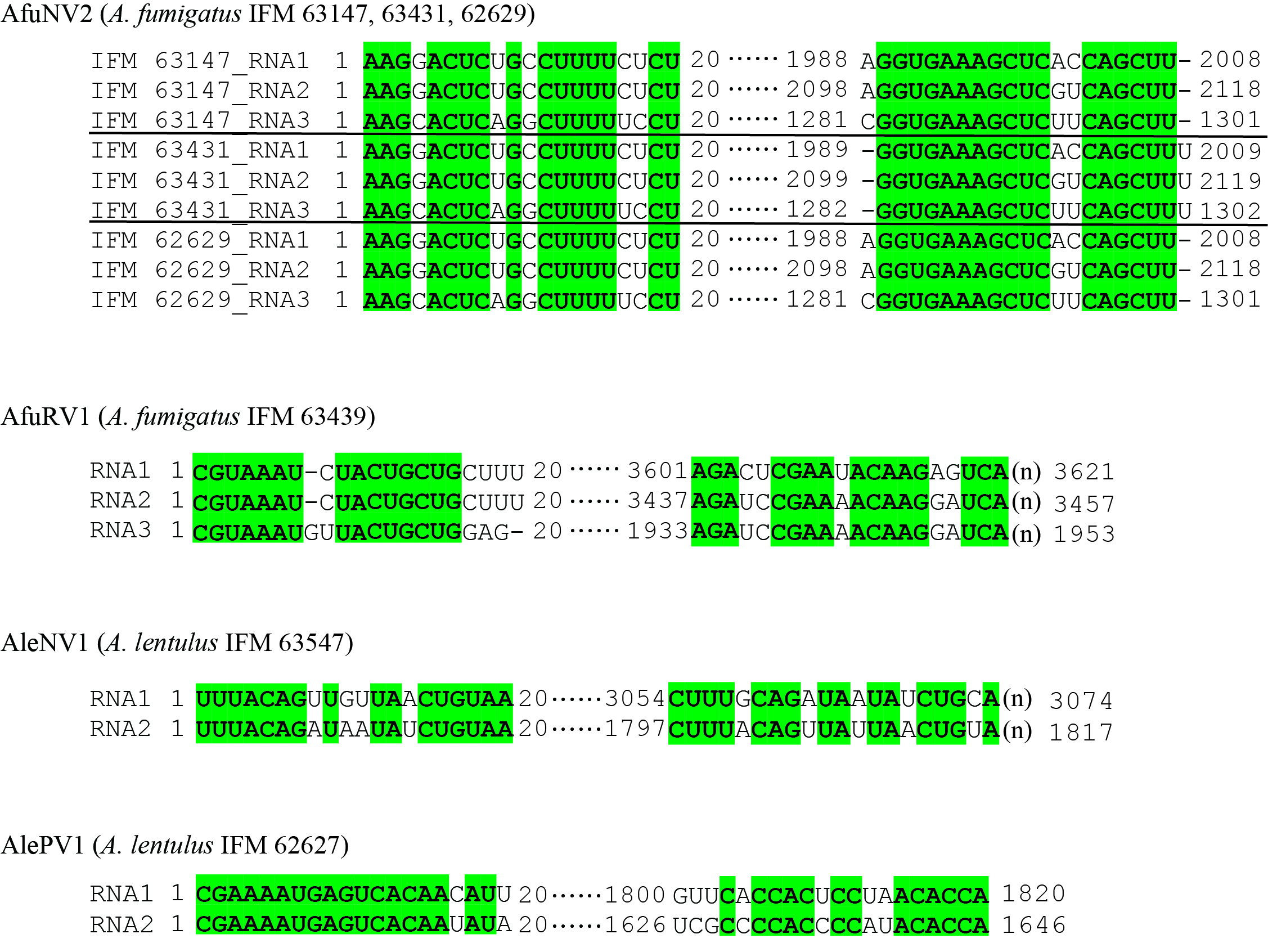

Supplement: veaa101_Supplementary_Data [file veaa101_Supplementary_Data.zip › Fig. S3.jpg]

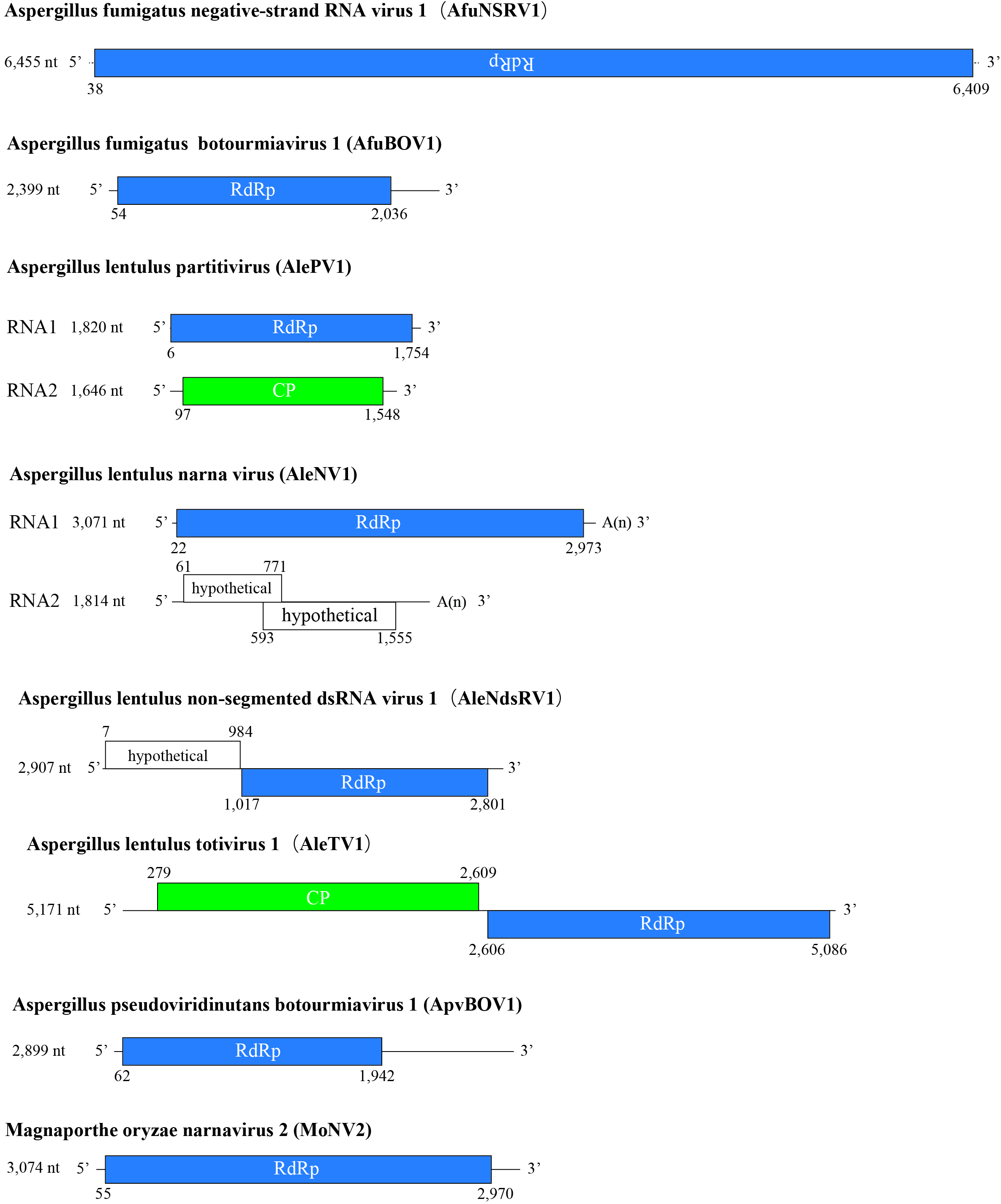

Supplement: veaa101_Supplementary_Data [file veaa101_Supplementary_Data.zip › Fig. S4.jpg]

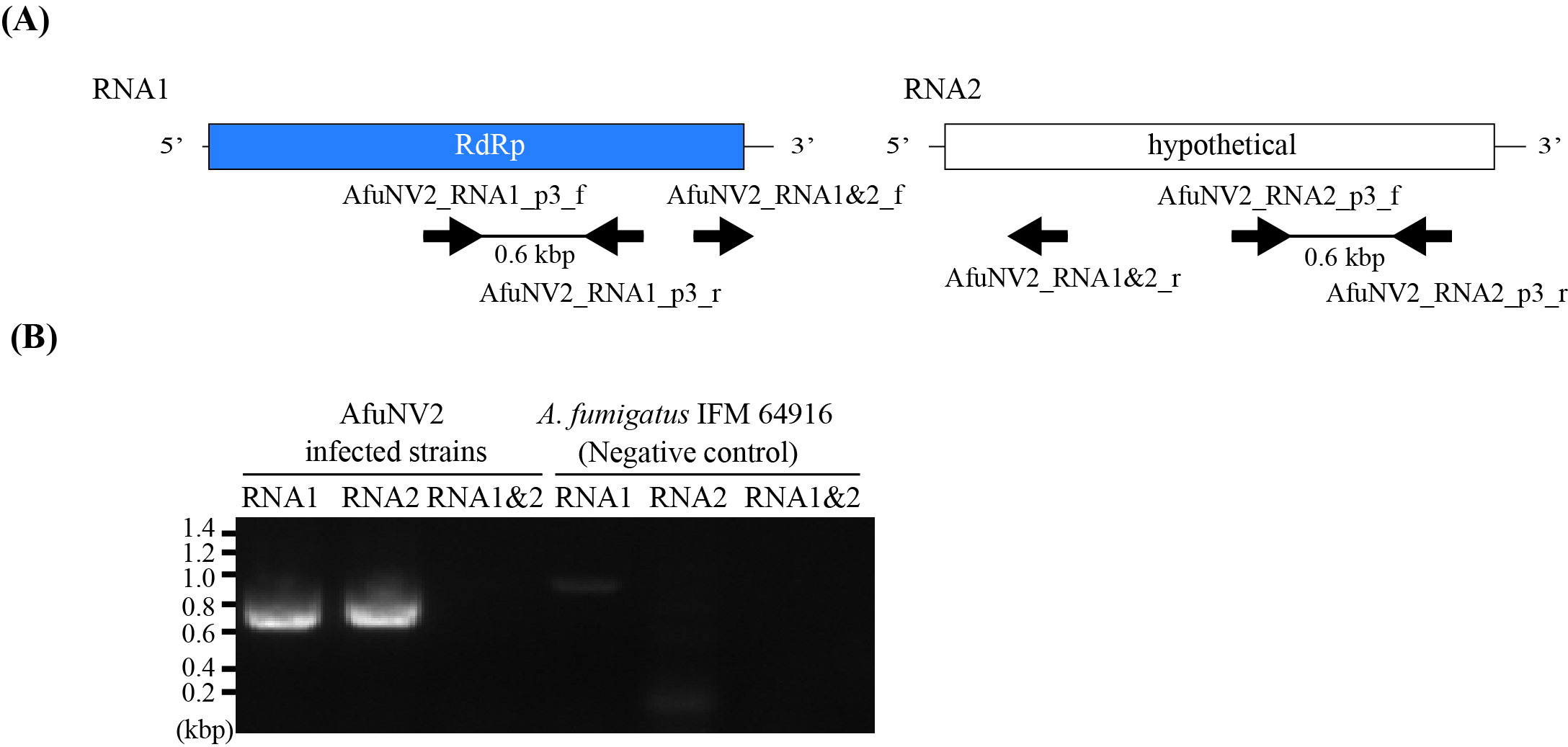

Supplement: veaa101_Supplementary_Data [file veaa101_Supplementary_Data.zip › Fig. S5.jpg]

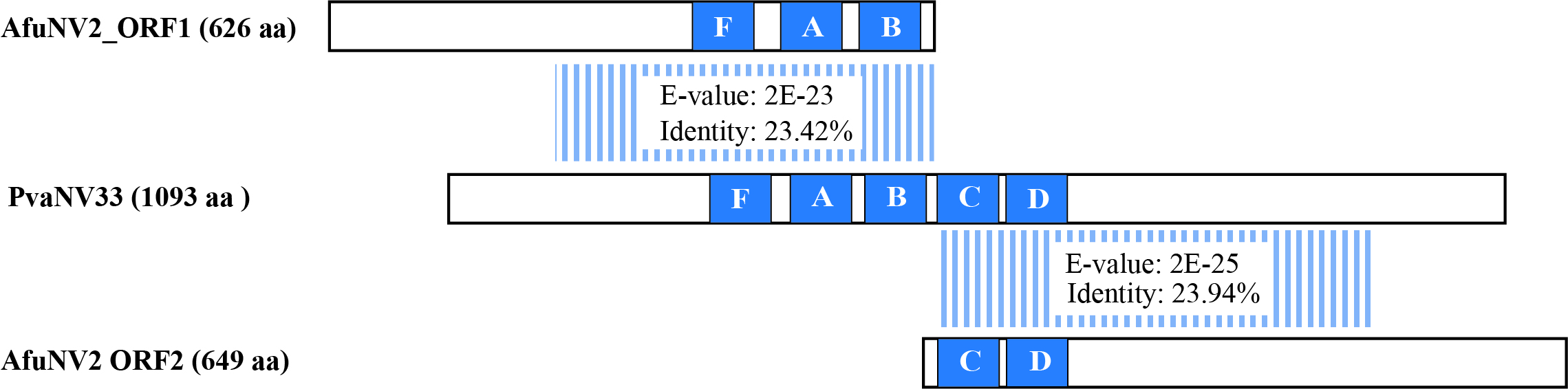

Supplement: veaa101_Supplementary_Data [file veaa101_Supplementary_Data.zip › Fig. S6.jpg]
